# Pain and self-rated health among middle-aged and older Canadians: an analysis of the Canadian community health survey―healthy aging

**DOI:** 10.1186/s12889-018-5912-9

**Published:** 2018-08-13

**Authors:** Batholomew Chireh, Carl D’Arcy

**Affiliations:** 1 University of Saskatchewan, School of Public Health, 104 Clinic Place, Saskatoon, SK S7N 2Z4 Canada; 2 Department of Psychiatry, College of Medicine, School of Public Health Saskatchewan Research Data Centre, University of Saskatchewan, Royal University Hospital, 103 Hospital Drive, Saskatoon, SK Canada

**Keywords:** Aging, Pain, Self-rated health, Epidemiology

## Abstract

**Background:**

Pain is an important health problem adversely affecting functionality and quality of life. Though self- rated health (SRH) is a major predictor of mortality, its relationship with pain is not well understood. We explore 1) how pain and age interact to influence SRH, and 2) provincial variations in SRH across Canada.

**Methods:**

We analyzed cross-sectional data from Statistics Canada’s Canadian Community Health Survey-Healthy Aging (*n* = 30,685), which targeted those 45 years and older and was conducted from 2008 to 12-01 to 2009–11-30. The response rate was 74.4%.The topics covered included socio-demographics, well-being and chronic diseases. We performed both bivariate and multivariate analyses between each predictor and SRH; unadjusted and adjusted odds ratios and 95% confidence intervals are reported. Two-level logistic regression mixed model was used to account for provincial differences. An intraclass correlation coefficient was also computed.

**Results:**

Slightly more than half of respondents (56.40%) were female. In bivariate analyses, those experiencing pain had an odds ratio of 0.20. Which means that the odds of reporting good self-rated health are 4 to 5 times lower for those with pain, compare to the odds of reporting good self-rated health among those without pain (*p* < 0.001). In multivariate analyses the highly educated, female gender, the never married or single and households with high yearly income were predictors of good health (*p* < 0.001). Those who reported depressive symptoms, the lonely, the obese, daily smokers and/or the stressed were less likely to rate their health as good (*p* < 0.001). The influence of pain on SRH was stronger among younger age groups (45–54 years) compared to older age groups (75-84 years, with an odds ratio of 3.53 [*p* < 0.001] versus 3.14 [p < 0.001]).

**Conclusions:**

Pain, among other determinants, is associated with SRH. Individuals in rating their health may consider a variety of factors, some of which may not be apparent to health providers. We found that those who reported depressive symptoms, were daily smokers, the obese, the lonely, and/or having a stressful life were less likely to rate their health as good. No significant provincial variations in SRH in Canada was observed in this study.

## Background

The world today is in a period of substantial change. Historically the world had high birth and mortality rates, high rates of infectious diseases and small stable populations. In the ensuing epidemiological transition improvements in sanitation and infection control has led to declining mortality and fertility rates. Populations both grew and became older. Aging populations are facts in both the developed and less developed countries. Global aging is now most responsible for morbidity and higher incidence of pain and disability [1].

Pain is an important public health problem that is shown to be negatively associated with a number of health outcomes and affects quality of life [2].It is also a major reminder of poor health in human populations. A study reported body places of pain location to include; the upper and lower limbs, the back (lumbar region), neck and joints, the face, abdomen, knee, hip, chest and rectum [3]. Incidence of pain symptoms have also been associated with increased health care utilization and costs [3]. A number of studies have shown that pain varies across demographic, economic and social characteristics [4–7]. Other studies also reported that pain prevalence decrease after middle-age [4, 5]. Evidence suggest that, whereas certain groups may experience pain more frequently than others, population characteristics, such as age, race and sex tend to moderate the way people report pain [8–10]. The above population characteristics makes the reporting of pain, partly psychosocially determined.

A few earlier studies found an association between pain and self-rated health [11–13]. A study by Reyes-Gibby et al. [11] found a negative association between persistent pain and self-assessed health in adults aged 70+ using data from the Asset and Health Dynamics among the Oldest Old Data (AHEAD). Another study in Finland comparing two age cohorts (age 15–44 and age 45–74) found chronic pain to be strongly associated with the younger age cohort compared to the older ages [12]. Rubin & Zimmer [14] found a robust association between pain and self-rated health and that the association continues to be stronger after adjusting for demographic, socio-economic and social support variables. A study conducted in Canada reported an association between poorer SRH and pain particularly a prolonged period of living with it [15].

Self-rated health (SRH) can be defined as an individual’s subjective condensed summary of information about his or her bodily conditions. In other words it is a statistical (rather than a causative) predictor of mortality. Self- rated health (SRH) is an important independent predictor of mortality [16]. SRH is also considered as a measure of general health status globally [17]. It is mostly used as a predictor of health outcomes in patient populations in areas such as use of health care resources, morbidity and mortality and recovery after disease [16, 18, 19]. In addition, most population based studies also use SRH as an outcome measure [20].

A number of determinants or predictors of self-rated have been examined in the literature.

For instance some studies examined the relationship between age and self-rated health and found that older persons tend to provide more favorable rating about their health compared to younger persons [21, 22]. In the same vain Shooshtari. et al. [23] found self-rated as a multidimensional concept and that age variations exist in the determinants of positive and negative self-rated health for younger and older Canadian adults. Layes et al. [24] found that self-rated health vary by age and socioeconomic status and that the oldest-old (80+ years) and those with less income and education were more likely to report positive health status or report optimism about their health. A study by Idler, E., & Cartwright, K. [25] also reported gender and race as important predictors of self-rated health and that younger respondents were more likely to poorly rate their health compared to cohorts of older adults. Depressive symptom as a predictor of SRH was reported in earlier studies [13, 26, 27].Other predictors such as physical functioning, exercise, life satisfaction and positive affect were found to be positively associated with SRH [27].

The importance of pain and age as determinants or predictors of self-rated health has been well documented in the literature. Most cross-sectional and longitudinal studies focused on the independent effects of age and pain on self-rated health. However, what is not well established is the interactive effect of the two predictors on SRH. In the Canadian context there has been little research to date that linked the interactive effect of pain and age on self-rated health. Also, the influence of geographic (provincial) variations on SRH in the Canadian context is not well explored. The objectives of this study are to explore 1) how pain and age interact to influence SRH, and 2) provincial variations in SRH across Canada.

The abstract of this article is present on a university repository website and can be accessed on https://www.canada.ca/en/public-health/services/reports-publications/health-promotion-chronic-disease-prevention-canada-research-policy-practice/vol-36-no-11-2016/cseb-student-conference-2016-abstract-contest-winners.html#a4. This article is not published nor is under publication elsewhere.

## Method

### Study population and data sources

We analyzed cross-sectional data from Statistics Canada’s National Canadian Community Health Survey**–**Healthy Aging (*n* = 30,685), which targeted those 45 years and older and was conducted from 2008 to 12-01 to 2009–11-30. The response rate was 74.4%. The topics covered included socio-demographics, well-being and chronic diseases. The exclusion criteria from the sampling frame was as follows; full-time members of the Canadian Forces and residents of the three territories, Indian reserves, Crown lands, institutions, and some remote areas. The survey was weighted to represent the population of 45 years of age and over living in the ten provinces of Canada between 2008 and 2009 [17].

### Measures

#### Main outcome variable

Self-rated health (SRH), the main outcome variable in this study, was measured by asking respondents the following question: “In general, would you say your health status is……” (Excellent, very good, good, fair or poor)? Categories of excellent, very good and good were collapsed and re-coded as one (1) and renamed (Good Health). All other categories such as fair/poor were re-coded as one (0) to reflect (Poor health) with ‘Poor Health’ as the reference category. Missing responses were dropped during recoding.

#### Primary exposure variable

Presence of self-reported pain was the primary exposure variable. It was measured on a 5-point scale on which respondent’s self-reported presence or absence of pain was reported. Pain levels were; 1 = “no pain”, 2 =“ pain does not prevent any activities”, 3= “pain prevents few activities”, 4= “pain prevents some activities”, 5= “pain prevents most activities” and “not stated”. The various levels of pain were dichotomised into yes and no categories whereby pain level 1 stood alone as “No pain” and pain levels 2 to 5 were collapsed to form a category of “Pain”. The “Not stated” category was treated as missing values and such cases dropped in subsequent analysis of the data.

#### Covariates

The socio-demographic, behavioral, and physical covariates entered in this analysis were: province of residence, area of residence (rural vs urban residence), gender, age (45–54, 55–64, 65–74, 75–84, > = 85), educational level (less than secondary, secondary graduation, other post-secondary and post-secondary graduation), marital status (never married, divorced/separate/widowed, married/ common law), total household annual income from all sources (<= $20,000, $20,000–$39,999, $40,000–$59,999, $60,000–$79,999, $80,000 or more and Not stated), body mass index (BMI) derived from self-reported height and weight, (underweight 16–18.5 kg/m^2^, normal weight 18.5–25 kg/m^2^, overweight 25–29 kg/m^2^ and obese > 30 kg/m^2^). Respondent’s smoking status was also assessed by asking them to indicate the type of smoker they were. Categories include, never smoked, former smoker, occasional and daily smoker. Categories of never smoked, former smoker and occasional smoker were recoded as zero (0) and named as never or former smoker while category of daily smoker stood alone as one (1) with the never or former smoker category as the reference group.

Three psychological covariates were also included in the analysis. Perceived life stress, was assessed by asking the question, “Thinking about the amount of stress in your life, would you say that most days.......” (Not at all stressful, not very stressful, a bit stressful, quite a bit stressful and extremely stressful)? The first two categories of “not at all” and “not very” were merged and recoded as (“no stress”), vs “a bit”, “quite a bit” and “extremely” (“stress”). Other categories such as “don’t know” and “refusal” were treated as missing values and left out of the analysis.

Depressive symptom was assessed by asking respondents this question: “During the past 12 months, was there ever a time when you felt sad, blue or depressed for two weeks or more in a row?” The following were responses to the question: “yes”, “no”, “don’t know”, “refusal” and “not stated”. We re-categorised depressive symptom into Yes = “1” and No = “0” and other values were treated as missing.

Loneliness was assessed on the following questions: “How often do you feel: that you lack companionship?” “left out?” “isolated from others?” The 6-point response scale was categorized as “Everfelt leftout/ loneliness” and hardly ever as “Neverfelt leftout/no loneliness” with “no loneliness” as reference group. Don’t know, refusal and not stated were regarded as missing data.

#### Statistical analysis

The frequencies and cross-tabulations were calculated by socio-demographic factors such as age, rural/urban residence, gender, marital status, household income and education. Taking into account sampling employed in the survey and the resulting hierarchical structure of the data, a two-level mixed model logistic regression with provincial differences (first level) and individual differences (second level) was used. This provides a more useful opportunity to measure the contextual effect of provincial differences in the association between the interactive effect of pain with age and SRH in this study.

The error term was used at the first level to account for the variation in the probability of reporting SRH among respondents in the different provinces across Canada. Univariate analyses between each predictor and outcome (SRH) in the two level models were performed and unadjusted odds ratios (UOR) and 95% confidence intervals (CI) were reported. Predictors with univariate values *p <* 0.20 were maintained for further use in the multivariate analysis [28].

In the multivariate model building we examined the association between predictor variables and the outcome. A manual backward elimination process was used to remove insignificant predictor variables one at a time starting with the variable with the highest *p-*value until variables with *p <* 0.05 were retained for subsequent analysis. All other variables with missing observations were removed from subsequent analysis. Potential confounders were also retained in the final model only if after including the variable it changes the coefficients of other variables or the primary predictor variable by > 10%. Interaction effects were also assessed by age and pain as well as pain with area of residence. Interactions that recorded *p <* 0.05 were regarded as significant. We used pairwise comparisons to assess differences across multiple categories of age groups and pain.

An intraclass correlation coefficient was computed to determine the degree of variability regarding respondents SRH at the provincial level by the use of the following statistical formula: ƿ = Ơ^2^ group / (Ơ^2^ group + π2/3). The adequacy of the final model was assessed using standardised residuals for fixed effects only at the individual level where no outliers were detected. Standardised residuals for random effects only were used to assess differences that might exist at the provincial level. With the exception of Saskatchewan that was an outlier, all other provinces fell within the acceptable range.

The overall significance of this logistic regression model was checked after all the predictors were included in the model without the interaction term and compared to the final model with the interaction term and a likelihood ratio test performed which produced a statistically significant value *(p = 0.0015).* Statistical analyses were completed in Stata 13 [29].

## Results

### Characteristics of the study population

Table 1 shows the demographic characteristics of study participants. The CCHS-HA study has a representative sample of 30,865 Canadians aged 45 + .We analyzed observations with complete data of 28,800 in our study. Data collection occurred during 2008–2009. The largest segment of the survey population was made up of those who fall within 55–64 years age group, women, married or in common law unions, having post-secondary graduation education, living in an urban area, with low annual household income, without pain, without depressive symptoms, never or former smokers, not stressed, with normal weight and with good SRH.

**Table 1:** Characteristics of respondents (aged 45+) covered in the 2008/2009 CCHS-HA survey

Characteristics	Frequency	Percentage (%)
Gender		
Male	12,557	43.60
Female	16,243	56.40
Total	28,800	100
Age categories, years		
45–54	4898	17.01
55–64	8875	30.82
65–74	6604	22.93
75–84	4925	17.10
85+	3498	12.15
Total	28,800	100
Educational level		
Less than secondary	9001	31.25
Secondary graduation	4718	16.38
Other post-secondary	1473	5.11
Post-secondary graduation	13,608	47.25
Total	28,800	100
Marital Status		
Married/common law	16,288	56.56
Divorced/separate/widowed	10,359	35.97
Never married	2153	7.48
Total	28,800	100
Self-reported BMI		
Normal weight	11,413	39.63
Underweight	524	1.82
Overweight	10,798	37.49
Obese	6065	21.06
Total	28,800	100
Place of residence		
Urban	16,148	56.07
Rural	12,652	43.93
Total	28,800	100
Total household income, CAD		
< = $20,000	4349	15.10
$20,000–$39,999	6760	23.47
$40,000–$59,999	4609	16.00
$60,000–$79,999	3097	10.75
$80,000 or more	5397	18.75
Not stated	4588	15.93
Total	28,800	100
Self-rated health		
Poor health	5735	19.91
Good health	23,065	80.09
Total	28,800	100
Life stress		
No	13,861	48.13
Yes	14,939	51.87
Total	28,800	100
Presence of pain		
No	21,119	73.33
Yes	7681	26.67
Total	28,800	100
Depressive symptom		
No	24,742	85.91
Yes	4058	14.09
Total	28,800	100.00
Type of smoker		
Never/former smoker	24,633	85.53
Daily	4167	14.47
Total	28,800	100.00

### Association between predictor variables and self-rated health among respondents aged 45+ (univariable analysis)

Only those survey participants with complete data on all study variables (*N* = 28,800) were used in the subsequent univariate and multivariate analyses. Univariate association between predictor variables and the outcome are presented in Table 2. Not surprisingly good SRH decreased with age. In comparison to the middle-aged group (45 – 54 years) respondents in the young-old (65–74 years) and oldest-old (aged 85+ years) were less likely to report good self-rated health with significant odds ratios and confidence intervals of (OR = 0.56, *p* < 0.001) and (OR = 0.36, *p* < 0.001) respectively.

**Table 2:** Socio-demographic factors/predictors of self-rated health and unadjusted odds ratios (UOR) with corresponding 95% CI and *p*-values measured in a two-level logistic model

Variable	Odds ratio	95%CI		P-value
		Lower	Upper	
Gender				
Male	Ref. category			
Female	1.03	0.97	1.09	0.323
Age categories, years				
45–54	Ref. category			
55–64	0.75	0.67	0.83	< 0.001
65–74	0.56	0.50	0.62	< 0.001
75–84	0.37	0.34	0.41	< 0.001
85+	0.36	0.32	0.40	< 0.001
Educational level				
Less than secondary	Ref. category			
Secondary graduation	2.21	2.02	2.42	< 0.001
Other post-secondary	2.12	1.84	2.45	< 0.001
Post-secondary graduation	2.57	2.40	2.74	< 0.001
Marital Status				
Married/common law	Ref. category			
Divorced/separate/widowed	0.66	0.62	0.70	< 0.001
Never married	0.75	0.67	0.83	< 0.001
Total household income, CAD				
< = $20,000	Ref. category			
$20,000–$39,999	1.52	1.40	1.65	< 0.00 1
$40,000–$59,999	2.52	2.28	2.79	< 0.001
$60,000–$79,999	3.74	3.30	4.24	< 0.001
$80,000 or more	5.72	5.09	6.42	< 0.001
Not stated	1.79	1.63	1.97	< 0.001
Place of residence				
Urban	Ref. category			
Rural	0.84	0.79	0.90	< 0.001
Presence of pain				
No	Ref. category			
Yes	0.20	0.19	0.21	< 0.001
Life stress				
No	Ref. category			
Yes	0.57	0.54	0.60	< 0.001
Self-reported BMI				
Normal weight	Ref. category			
Underweight	0.43	0.36	0.52	< 0.001
Overweight	0.96	0.89	1.03	0.226
Obese	0.60	0.56	0.65	< 0.001
Depressive symptoms				
No	Ref. category			
Yes	0.45	0.42	0.49	< 0.001
Loneliness				
No	Ref. category			
Yes	0.43	0.40	0.46	< 0.001
Type of smoker				
Never/former smoker	Ref. category			
Daily	0.66	0.61	0.71	< 0.001

SRH was positively related to education. Compared to the less educated participants, those who had post-secondary graduation were (OR = 2.57, *p* < 0.001) times more *likely* to rate their health as good. Similarly SRH was positively associated with income, those in the highest household income bracket, ($80,000+) income, were (OR = 5.72, *p* < 0.001) times more likely to report good SRH compared to the reference group (<= $20,000). The higher your household income level, the more likely you were to report good health.

There was a negative association between BMI and SRH. Obese respondents were (OR = 0.60, *p* < 0.001) less likely to rate their health as good compared to those with normal weight. Rural dwellers were less likely to report good SRH compared to their urban counterparts (OR = 0.84, *p* < 0.001). Our univariable analysis found that females were more likely to rate their health as good compared to males (OR = 1.03, *p* = 0.323).

Those experiencing daily pain reported an odds ratio of 0.20. This means that the odds of reporting good self-rated health are 4 to 5 times lower for those with pain, compare to the odds of reporting good self-rated health among those without pain (*p* < 0.001). Other factors including life stress, loneliness, depressive symptoms and smoking status were negatively associated with good SRH (*p* < 0.001). All the predictors examined at the univariate analysis except gender were associated with SRH, (*p* < 0 .001).

### Predictors of self-rated health among respondents aged 45+ (a two level multivariate analysis)

Rural/urban residence was removed from the model during the backward selection process due to an insignificant value (*p* = 0.54). An interaction between pain and area of residence was subsequently examined which produced an insignificant value (*p =* 0.57) and was removed from the model. We however found an interaction between age and pain *(p <* 0.001*)*. All the variables with values *(p <* 0 .20*)* in the initial univariate analyses were forwarded for multivariate logistic regression modeling. Table 3 represents the final two level multivariate logistic regression model for SRH among those aged 45 years and over after adjusting for covariates.

**Table 3:** A final two-level multivariate model without interaction terms measuring association between predictor variables and self-rated health with corresponding adjusted odds ratios,95% CI, and *p*-values

Variable	Odds ratio	95%CI		P-value
		Lower	Upper	
Gender/sex				
Male	Ref. category			
Female	1.36	1.28	1.46	< 0.001
Educational level				
Less than secondary	Ref. category			
Secondary graduation	1.82	1.66	2.01	< 0.001
Other post-secondary	1.83	1.58	2.13	< 0.001
Post-secondary graduation	1.98	1.84	2.13	< 0.001
Total household income, CAD				
< = $20,000	Ref. category			
$20,000–$39,999	1.35	1.23	1.47	< 0.001
$40,000–$59,999	2.06	1.84	2.31	< 0.001
$60,000–$79,999	2.99	2.60	3.43	< 0.001
$80,000 or more	4.43	3.88	5.07	< 0.001
Not stated	1.45	1.30	1.60	< 0.001
Marital status				
Married/common law	Ref. category			
Divorced/separate/widowed	1.03	0.95	1.10	0.494
Never married/single	1.19	1.05	1.34	0.005
Life stress				
No	Ref. category			
Yes	0.59	0.55	0.63	< 0.001
Self-reported BMI				
Normalweight	Ref. category			
Underweight	0.48	0.39	0.59	< 0.001
Overweight	0.93	0.86	0.99	0.046
Obese	0.60	0.55	0.65	< 0.001
Depression				
No	Ref. category			
Yes	0.59	0.54	0.64	< 0.001
Loneliness				
No	Ref. category			
Yes				
Type of smoker	0.58	0.54	0.63	< 0.001
Never/former smoker	Ref. category			
Daily	0.79	0.73	0.86	< 0.001

There was a positive relationship between educational level and SRH. In comparison with the lowest level of education, the adjusted odds of reporting good SRH were (OR = 1.82, *p* < 0.001) for the secondary graduation, (OR = 1.83, *p* < 0.001) for other post-secondary graduation and (OR = 1.98, *p* < 0.001) for post-secondary graduation respectively. Implying that the higher the educational level of respondents, the higher the odds of reporting good self-rated health.

Also, those who fall within the highest household income bracket ($80,000+) income (OR = 4.43, *p* < 0.001) had higher odds of reporting good SRH compared to those in the lowest household income group. Those that were obese (OR = 0.60, *p* < 0.001) and overweight (OR = 0.93, *p* < 0.001) were less likely to rate their health as good compared to normal weight respondents.

Respondents who were daily smokers, had depressive symptoms, lonely and those who were stressed were less likely to rate their health as good (*p* < 0.001). Respondents that were single or never married were (OR = 1.19, *p* = 0.005) times more likely to rate their health as good. Females were (OR = 1.36, *p* < 0.001) times more likely to report good self-rated health compared to their male counterparts. The present study produced an intraclass correlation coefficient (ICC) of 0.32% in the final two level model indicating that, no strong variations exist in respondent’s SRH at the provincial level.

### Measuring the interactive effect of age and pain on self-rated health taken into account effect of other covariates

The difference in the odds of reporting good SRH among age groups is based on the presence or absence of pain. Our study reported an odds ratio of (OR = 0.84, *p* = 0.012) in the oldest-old group (> = 85 years- no pain) where pain is completely absent. It means that the odds of reporting good self-rated health is 16% lower in the oldest-old group compared to 35% in the middle-aged group. Which implies that, in the absence of pain, senior are better positioned to rate their health as good compared to middle-aged respondents.

In addition, in the presence of pain, in the oldest-old age group (> = 85 years pain) our study found an odds ratio of (OR = 0.68, *p* < 0.001). This means that the odds of reporting good self-rated health is 32% lower in the oldest-old group compared to 23% in the middle-aged group. This therefore means that, middle-aged respondents are able to favorably rate their health even in the presence of pain compared to oldest-old group.

We examined the importance of (no-pain vs pain) and their influence on SRH in our study. We found that in the absence of pain in one group and presence of pain in the other group, pain shows more importance. For instance, we found that compared to the middle-aged group with pain (45-54 years-pain), respondents without pain in the oldest-old age group (> = 85 years-no pain) were 1.35 times more likely to rate their health as good (*p* < 0.001). Also, respondents in the young-old group without pain (65-74 years-no pain), were 2.39 times more likely to rate their health as good compared to the middle-aged group (45-54 years-pain) with pain (*p* < 0.001). Table 4 summarized the interaction between age and pain. The results show that pain has much influence on respondent’s SRH than age. This was clearly evident in the comparison among age categories for those without and with pain.

**Table 4:** A pair wise comparison of the interactive effect of pain and age and its influence on self-rated health from a two-level multivariate model with corresponding odds ratios, 95% CI, and *p*-values

Comparison	OR	95%CI		P-value
		Lower	Upper	
Differences among age categories for those without pain				
55-64 years- no pain vs 45-54 years-no pain	0.65	0.55	0.77	< 0.001
65-74 years- no pain vs 45-54 years-no pain	0.44	0.37	0.52	< 0.001
75-84 years- no pain vs 45-54 years-no pain	0.30	0.25	0.35	< 0.001
> =85 years- no pain vs 45-54 years-no pain	0.25	0.21	0.30	< 0.001
65-74 years- no pain vs 55-64 years- no pain	0.68	0.60	0.77	< 0.001
75-84 years- no pain vs 55-64 years- no pain	0.46	0.40	0.52	< 0.001
> =85 years- no pain vs 55-64 years- no pain	0.38	0.33	0.44	< 0.001
75-84 years- no pain vs 65-74 years- no pain	0.67	0.60	0.76	< 0.001
> =85 years- no pain vs 65-74 years- no pain	0.57	0.49	0.65	< 0.001
> =85 years- no pain vs 75-84 years- no pain	0.84	0.73	0.96	0.012
Differences among age categories for those with pain				
55-64 years-pain vs 45-54 years-pain	0.77	0.66	0.91	0.002
65-74 years-pain vs 45-54 years-pain	0.64	0.54	0.76	< 0.001
75-84 years-pain vs 45-54 years-pain	0.43	0.36	0.52	< 0.001
> =85 years-pain vs 45-54 years-pain	0.43	0.35	0.53	< 0.001
65-74 years-pain vs 55-64 years-pain	0.82	0.72	0.95	0.006
75-84 years-pain vs 55-64 years-pain	0.56	0.48	0.65	< 0.001
> =85 years-pain vs 55-64 years-pain	0.56	0.47	0.66	< 0.001
75-84 years-pain vs 65-74 years-pain	0.68	0.58	0.79	< 0.001
> =85 years-pain vs 65-74 years-pain	0.68	0.57	0.80	< 0.001
> =85 years-pain vs 75-84 years-pain	0.99	0.85	1.18	0.997
Difference between those without and with pain for each age category				
55-64 years-no pain vs 45-54 years-pain	3.53	3.01	4.14	< 0.001
65-74 years- no pain vs 45-54 years-pain	2.39	2.03	2.82	< 0.001
75-84 years- no pain vs 45-54 years- pain	1.61	1.36	1.91	< 0.001
> =85 years-no pain vs 45-54 years- pain	1.35	1.13	1.63	0.001
65-74 years- no pain vs 55-64 years-pain	3.09	2.72	3.51	< 0.001
75-84 years- no pain vs 55-64 years- pain	2.08	1.82	2.38	< 0.001
> =85 years-no pain vs 55-64 years- pain	1.75	1.51	2.03	< 0.001
75-84 years-no pain vs 65-74 years- pain	2.53	2.20	2.90	< 0.001
> =85 years-no pain vs 65-74 years-pain	2.12	1.82	2.47	< 0.001
> =85 years- no pain vs 75-84 years-pain	3.14	2.70	3.65	< 0.001

Also, the results of the (no-pain vs pain) comparison showed that pain was more problematic to the middle-aged compared to the oldest-old. A possible explanation to this finding could be the fact that the middle aged and the young-old are still active and are most likely to engage in activities or job related occupations that are physically demanding and can result in pain. Pain also interferes with the expectations and activities of middle-aged and young-old individuals.

## Discussion

This study used data from a nationally representative population of middle aged and older Canadians to determine the influence of the combined effect of age and pain in assessing how respondents subjectively rated their state of health. We also assessed differences in self-rated health across the provinces.

We found in our no pain and pain comparison that the effect of pain on SRH decreases with increasing age suggesting that pain is more problematic to the middle-aged and young-old adults compared to older adults. This is consistent with previous studies where pain prevalence in specific parts of the body declines with advancing age [4, 5, 30]. Our study however contradicts an earlier study which suggests pain increases with age [31]. Our present finding suggests that older seniors maybe more accepting to pain and may see some degree of pain as “normal”. It can also be as a result of differences in health expectations at different ages. Another possible explanation is that older persons have higher expectations of experiencing pain, and this may diminish their experience of pain as an important determinant of global health.

Our finding on the interactive effect of pain and age shows that pain has more influence on SRH than age and that older adults without pain tend to rate their health as good compared to younger adults without pain. This is in line with previous studies [21, 22, 25] . In a study by Idler, E., & Cartwright, K. [25] for example they found that older age was related to better health rather than poorer health, once other factors are accounted for. It however contradicted earlier studies that showed age as an important moderator in the pain and SRH relationship. [8–10].

We found that higher income was positively associated with SRH. Previous studies showed a positive association between income and SRH [23, 32]. Our results also showed income as a major positive determinant of health even though it contradicts Layes et al. [24] who found lower income status to be associated with positive SRH. A possible explanation to our finding is that wealthy persons are more likely to have access to programs and services that exist in their places of residence and this may promote their health and help them to be more positive about their overall health status.

In our study higher education was positively associated with SRH. This confirms previous studies where higher educational levels were associated with positive SRH [22, 33]. Our finding maybe explained by the likelihood of more educated people to be better informed about their health status, their access to better medical care and generally their ability to live comfortable lives.

We found that females were more likely to report good SRH than males. This finding can be due to differences in health seeking behaviours and changes in risk behaviours between men and women. The association can also be explained by women’s increased knowledge of morbidity than men and their familiarity with aches and pains. Our study confirms earlier findings in the association between gender and SRH. [22, 25].

As expected, respondents who were overweight or obese were less likely to rate their health as good. Our finding is in the expected direction and is also consistent with a previous study that found obesity and overweight to be associated with a decrease odds of positive SRH [23]. This finding however disagrees with a finding reported by Simon and colleagues in 2005, who concluded that health behaviour and lifestyle factors are not important in health assessments [34].

We found marital status (single or never married status) to be significantly positively associated with good SRH. Our finding disagrees with Shooshtari. et al. [23] who found no significant association between a number of social environment factors including marital status among older Canadian adults. Respondents who experienced depressive symptom also had a lower odds of rating their health as good. This is in keeping with previous studies that found depressive symptom as a predictor of SRH [13, 26, 27]. Vivian et al. [35] in a hospital-based study in Taiwan found a significant association between patients with depression, life-time suicide ideation, life-threatening events and metastatic cancer and poorer SRH.

There was a strong association between perceived life stress and SRH in our study which is consistent with previous literature [36]. Loneliness was found to be negatively associated with SRH. In both univariate and multivariate analysis, those who were lonely were less likely to rate their health as good. This confirms previous research that found loneliness to be associated with poor SRH [37, 38].

We also found that daily smokers were 21% less likely to rate their health as good compared to non-smokers. This is in keeping with findings from a recent longitudinal study of adults (aged 50–104 years) where smoking status was associated with poor self-rated and predicted mortality at each time point [39].

The contributions of contextual and compositional factors as social determinants of health cannot be over emphasized. We did not find any significant provincial variations in SRH in our study. Our finding contract what earlier studies found. For example, Layes et al. [24] in a Canadian study examining geographic and language differences found that, Anglophones are more optimistic about their health compared to pessimistic Francophones. Another Canadian study however found individual-level factors to be more important than contextual factors [40]. Two national studies in England and Finland found a significant relationship between self-rated health and neighbourhood socio-economic attributes [41, 42].

### Strengths and limitations

The strength of the current study is the use of a large, population-based survey of the Canadian population, with an excellent response rate. The CCHS-HA data provides comprehensive data on descriptive variables, enabling in-depth analysis of the self-rated health among middle-aged and older adults. The present study is original in exploring provincial and individual level differences in SRH across Canada. Examining the interactive effect of pain and age on SRH is another strength of this study. This study has a number of limitations. Firstly, due to the cross-sectional nature of the CCHS-HA study, causality cannot be inferred. Secondly, the CCHS-HA study does not include respondents living in nursing homes and in Canada’s northern territories which may bias the results. Also, Prince Edward Island had no data on urban residents this limited some of the analyses.

## Conclusion

The current study compared self-rated health among different age categories among Canadian middle-aged and older adults and explored how pain and age interact to influence respondent’s self-rated health and other associated predictors. Predictors such as loneliness, depressive symptoms, life stress, smoking status and BMI were significantly negatively associated with SRH. High household income, higher educational attainment, marital status (single or never married status) and female gender stand out as major determinants of SRH. Targeted health care delivery should be carried out to reduce health inequalities. A longitudinal study would help establish causality for the factors found associated with SRH in this and many other studies.

## Appendix

Final equation for a two level model of self-rated health among middle aged and older Canadians.

$$ {\displaystyle \begin{array}{c}\mathrm{Log}\frac{\left(\uppi \mathrm{i}\right)}{\left(1\hbox{-} \uppi \mathrm{i}\right)}=\upbeta 0+{\upbeta}_1{\mathrm{X}}_{\mathrm{pain}}+{\upbeta}_2{\mathrm{X}}_{\mathrm{age}}+{\upbeta}_3{\mathrm{X}}_{\mathrm{BMI}}+{\upbeta}_4{\mathrm{X}}_{\mathrm{Maritalstatus}}+{\upbeta}_5{\mathrm{X}}_{\mathrm{stress}}+{\upbeta}_6{\mathrm{X}}_{\mathrm{depression}}+{\upbeta}_7{\mathrm{X}}_{\mathrm{Household}\ \mathrm{i}\mathrm{ncome}}\\ {}+{\upbeta}_8{\mathrm{X}}_{\mathrm{education}}+{\upbeta}_9{\mathrm{X}}_{\mathrm{smoker}\ \mathrm{type}}+{\upbeta}_{10}{\mathrm{X}}_{\_\mathrm{IEDUC}\_2}+{\upbeta}_{11}{\mathrm{X}}_{\_\mathrm{IEDUC}\_3}+{\upbeta}_{12}{\mathrm{X}}_{\_\mathrm{IEDUC}\_4}+{\upbeta}_{13}{\mathrm{X}}_{\_\mathrm{IMaritalSt}\_2}+{\upbeta}_{14}{\mathrm{X}}_{\_\mathrm{IMaritalSt}\_3}\\ {}+{\upbeta}_{15}{\mathrm{X}}_{\_\mathrm{Iincome}\_2}+{\upbeta}_{16}{\mathrm{X}}_{\_\mathrm{Iincome}\_3}\kern0.5em {\upbeta}_{17}{\mathrm{X}}_{\_\mathrm{Iincome}\_4}\kern0.5em {\upbeta}_{18}{\mathrm{X}}_{\_\mathrm{Iincome}\_5}\kern0.5em {\upbeta}_{19}{\mathrm{X}}_{\_\mathrm{Iincome}\_6}+{\upbeta}_{20}{\mathrm{X}}_{\_\mathrm{IAGE}\_\mathrm{cat}\_2}+{\upbeta}_{21}{\mathrm{X}}_{\_\mathrm{IAGE}\_\mathrm{cat}\_3}\\ {}+{\upbeta}_{22}{\mathrm{X}}_{\_\mathrm{IAGE}\_\mathrm{cat}\_4}+{\upbeta}_{23}{\mathrm{X}}_{\_\mathrm{IAGE}\_\mathrm{cat}\_5}+{\upbeta}_{24}{\mathrm{X}}_{\_\mathrm{IBMI}\_2}+{\upbeta}_{25}{\mathrm{X}}_{\_\mathrm{IBMI}\_3}+{\upbeta}_{26}{\mathrm{X}}_{\_\mathrm{IBMI}\_4}+{\upbeta}_{27}{\mathrm{X}}_{\mathrm{age}\ast \mathrm{pain}}+\upmu \end{array}} $$

Where the subscripted names represents:

- Pain: self-reported presence of pain; reference category = no
- Rural/urban: rural/urban area of residence; reference category = urban
- Age: groups of age; reference category = 45-54 years
- BMI: self-reported body mass index; reference category = normal weight
- Stress: self-reported life stress; reference category = no
- Depression: self-reported depression status; reference category = no
- Household income: Household yearly income bracket; reference category = lowest
- Education: educational level; reference category = less than secondary
- Smoker type: self-reported smoking status; reference category = no smoker
- Age*pain: how age and pain interact to influence self-perceive health
- *μ* error at the provincial level
